# Bone and cartilage differentiation of a single stem cell population driven by material interface

**DOI:** 10.1177/2041731417705615

**Published:** 2017-05-15

**Authors:** Hannah Donnelly, Carol-Anne Smith, Paula E Sweeten, Nikolaj Gadegaard, RM Dominic Meek, Matteo D’Este, Alvaro Mata, David Eglin, Matthew J Dalby

**Affiliations:** 1 Centre for Cell Engineering, University of Glasgow, Glasgow, UK; 2 Division of Biomedical Engineering, University of Glasgow, Glasgow, UK; 3 Department of Orthopaedics, Southern General Hospital, Glasgow, UK; 4 AO Research Institute Davos, Davos, Switzerland; 5 Institute of Bioengineering, Queen Mary University of London, London, UK; 6 School of Engineering and Materials Science, Queen Mary University of London, London, UK

**Keywords:** Nanotopography, hydrogel, mesenchymal stem cells, complex tissue engineering, endochondral tissue engineering

## Abstract

Adult stem cells, such as mesenchymal stem cells, are a multipotent cell source able to differentiate towards multiple cell types. While used widely in tissue engineering and biomaterials research, they present inherent donor variability and functionalities. In addition, their potential to form multiple tissues is rarely exploited. Here, we combine an osteogenic nanotopography and a chondrogenic hyaluronan hydrogel with the hypothesis that we can make a complex tissue from a single multipotent cell source with the exemplar of creating a three-dimensional bone–cartilage boundary environment. Marrow stromal cells were seeded onto the topographical surface and the temperature gelling hydrogel laid on top. Cells that remained on the nanotopography spread and formed osteoblast-like cells, while those that were seeded into or migrated into the gel remained rounded and expressed chondrogenic markers. This novel, simple interfacial environment provides a platform for anisotropic differentiation of cells from a single source, which could ultimately be exploited to sort osteogenic and chondrogenic progenitor cells from a marrow stromal cell population and to develop a tissue engineered interface.

## Introduction

Articular cartilage, found at the surface ends of long bones, is an avascular, aneural connective tissue with a functional capacity to dissipate biomechanical loads and allow smooth articulation of joints.^1^ At the macroscopic level, it contains just one functional cell type, the chondrocyte. The cells regulate, synthesize and assemble a complex extracellular matrix (ECM) during the chondroblastic stage before terminally differentiating to the chondrocyte to maintain the tissue. However, due to the avascular nature of the tissue, repair is highly limited after injury and degradation is mainly irreversible leading to osteoarthritis (OA), which is a degenerative and disabling joint disease.

One of the main issues in repair of articular cartilage injury is that it often involves two tissues, bone and cartilage at their interface. Current treatments involving chondrocyte implantation to the defected site do not address the importance of the interface with the subchondral bone. Microfracture technique consisting of mobilizing cells from the bony region tends to be suboptimal in terms of cartilage tissue formation.

Thus, the use of stem cells, particularly skeletal marrow stromal cells (MSCs) capable of forming both cartilage and bone, may be desirable as follows: (a) they have high proliferative capacity and (b) their use would allow complex and interfacial tissue engineering (i.e. forming bone and cartilage) from a single cell type. However, distinct environmental cues are required to support differentiation down the desired osteo- or chondro-lineage of the MSCs. We note that while stem cells, particularly MSCs, are widely used in biomaterials and tissue engineering research, achieving spatial positioning of different cell types from a single cell source within a scaffold is challenging as different cell cues are required at different locations. Such cues can be physical (chemistry of the scaffold/grafting of chemistry, stiffness control, topographical information^2–7^), biological (i.e. peptides, growth factors^8^) or a combination of both.^9,10^ Growth factors have been added with spatial control to direct multiple stem cells fates.^11,12^ Similarly, plasmids were spatially delivered through a bilayer structure for the differentiation of MSCs towards bone and cartilage.^13^ While the use of a biphasic scaffold is not novel,^14^ the self-organization of hMSCs capabilities on designed scaffolds in basal media is both desirable and novel.

In this study, we employed an osteogenic nanoscale topography^6^ embossed on a biodegradable, poly(ϵ-caprolactone) (PCL) membrane and a hyaluronan hydrogel capable of supporting chondrogenesis.^15^ This modular system is designed to permit osteogenesis at the topography/gel interface and chondrogenesis within the gel using a multipotential, but critically single cell type source, MSCs, in the absence of directing external biological cues.

## Materials and methods

### Generation of FLAT and NSQ surfaces

As previously described, quartz slides of near-square (NSQ) topography (120 nm pits in square arrangement, centre–centre spacing of 300 nm, with ±50 nm offset in pit placement in x and y axes^4,6^) and glass coverslips of FLAT topography were used to create multiple polymer replicas by manual hot (80°C) embossing PCL beads ((C_6_H_10_O_2_)n; PCL (Sigma Aldrich, USA)): typical discs 13 mm in diameter, suitable for cell culture in 24-well plates. PCL discs were treated for 30 s at MHz-range radiofrequency (RF) in a plasma cleaner (PDC-002 Harrick Plasma) to remove organic contaminants and activate the surface to improve cell surface attachment, and then sterilized in 70% ethanol for 30 min and two sequential 5-min washes in cell culture media prior to cell seeding.

### Synthesis of poly(N-isopropylacrylamide) hyaluronan derivative

The derivative was prepared as already reported.^16^ Briefly, amino-terminated poly(*N*-isopropylacrylamide) (PNIPAM-NH_2_) was synthesized by dissolving 10 g of *N*-isopropylacrylamide in 20 mL of dry *N,N*-dimethylformamide (DMF). Under nitrogen atmosphere, 15 mg of azobisisobutyronitrile and 30 mg of cysteamine hydrochloride were added, and the reaction was let to proceed for 6 h. The product was precipitated and washed with diethyl ether. The PNIPAM-NH_2_ M_w_ value was 40,300 g/mol as measured by gel permeation chromatography. Hyaluronic acid (HA) sodium salt from *Streptococcus equi* (HANa) with M_w_ of 293,000 g/mol and polydis-persity (PDI) = 1.86 (Contipro Biotech, Czech Republic) was transformed in its tetrabutylammonium salt (HATBA) via cationic exchange. Then, 2.0 g of HATBA was dissolved in 200 mL of dry dimethyl sulfoxide at room temperature. Methanesulfonic acid and 1,1′-carbonyldiimidazole both equimolar to the repeating unit of HA were added, and 3.7 g of PNIPAM-NH_2_ added and stirred at room temperature for 3 days. The solution was dialysed against demineralized water using regenerated cellulose dialysis tubes (MWCO 50 kDa) for 5 days and finally freeze dried. ^1^H nuclear magnetic resonance (NMR) analysis was performed on a Bruker Avance AV-500 NMR spectrometer using deuterium oxide as solvent to assess the degree of grafting of pNIPAM-NH_2_ onto HA.

### Reconstitution and rheology of thermoresponsive hyaluronan solution

The hyaluronan derivative was reconstituted to 15% w:v in sterile phosphate-buffered saline (PBS) and stored at 4°C for 24 h for complete dissolution. Rheological measurements were performed on an Anton Paar MCR-302 rheometer equipped with Peltier temperature control device and thermostatic hood. A 1° conical geometry of 25 mm diameter and 49 µm gap was used. For each sample, an amplitude sweep was measured at 10 rad/s and 37.00 ± 0.03°C. Storage moduli were measured as function of the temperature between 20°C and 40°C with a gradient of 1°C/min at angular frequency of 10 rad/s and amplitude within the linear viscoelastic range. A thin layer of low-viscosity silicon oil was spread along the meniscus interface in order to avoid evaporation.

### Cell isolation and culture

MSCs were isolated from haematologically normal patients undergoing routine surgery as previously described.^17^ MSCs were cultured in growth media containing 86% Dulbecco’s Modified Eagle Medium (DMEM; Sigma, UK) supplemented with 10% fetal bovine serum (FBS) (Sigma), 2% penicillin streptomycin, 1% non-essential amino acids (Invitrogen, UK) and 1% 100 mM sodium pyruvate (Life Technologies, UK) at 37°C with a 5% CO_2_ atmosphere. Media was changed every 3 days and cells passaged to passage 2 or 3. Human mesenchymal stem cells (hMSCs) were seeded on NQS topography PCL surfaces in 24-well plates at 1 × 10^4^ cells per PCL disc (surface area of disc average 1.13 cm^2^), media as recipe above and incubated for 24 h to allow for adherence. Then, 250 µL of 1 × 10^4^ cells/mL of thermoresponsive hyaluronan composition at temperature around 10°C was added and allowed to flow on top of the PCL disc before incubation at 37°C for 10–15 min to allow gelation, the resultant gel is approximately 0.5 cm in thickness. The 13-mm glass coverslips sterilized in 70% ethanol were subsequently added on top to ensure that the hydrogel was always in contact with the underlying PCL disc. Note that MSCs from Promocell were used to generate Supplementary Figure 1.

### Immunocytochemistry

After 5 days of culture, cells were fixed (10 mL 37% formaldehyde, 2 g sucrose in 90 mL PBS solution) for 15 min. Permeabilizing buffer (10.3 g sucrose, 0.292 g NaCl, 0.06 g MgCl_2_, 0.476 g HEPES, 0.5 mL Triton X, in 100 mL of H_2_O, at pH 7.2) was then added for 15 min to control samples without hydrogel (−GEL), and for 2 h to samples with hydrogel (+GEL). To block non-specific binding, samples were incubated in 1% bovine serum albumin (BSA)/PBS for 15 min −GEL and 1 h +GEL. Primary antibodies (1:50 in 1% BSA/PBS) were added at 200 µL/well for 1 h (−GEL) and at 500 µL/well overnight (+GEL). Substrates were then washed three times in 0.5% Tween 20/PBS (5 min each –GEL, 20 min each +GEL). Corresponding secondary biotin-conjugated antibody (1:50 in 1% BSA/PBS) was added for 3 h to –GEL and +GEL samples, followed by substrate washing as described above. Fluorescein isothiocyanate (FITC)-conjugated streptavidin was added (1:50 in 1% BSA/PBS; Vector Laboratories, UK) for 2 h before samples were given a final wash. All immunostaining were carried out at 37°C with warmed solutions in order to maintain hydrogel integrity. Surfaces were mounted using mounting medium for fluorescence, with DAPI (4′,6-diamidino-2-phenylindole) counterstain (Vector Laboratories), and viewed by fluorescent microscopy (Zeiss Axiophot). Digital images were captured in two fluorescent channels (×20 magnification) and saved for further processing. Primary antibodies are presented in Table 1. Secondary antibodies are biotinylated monoclonal anti-mouse (IgG) raised in horse and fluorescein streptavidin (all Vector Laboratories).

**Table 1.** Primary antibodies used for immunocytochemistry.

Target molecule	Host	Isotype	Source	Reference
β3 tubulin	Mouse	Monoclonal, IgG2b	Sigma, UK	18–20
Phosphorylated RUNX2	Mouse	Monoclonal, IgG2a	Abcam, UK	21, 22
Vimentin	Mouse	Monoclonal, IgG1	Sigma, UK	34
SOX9	Mouse	Monoclonal, IgG2a	Abcam, UK	25, 26
Osteocalcin	Mouse	Monoclonal, IgG2a	Santa Cruz Biotechnology, USA	23, 27

### Von Kossa staining

After 28 days of culture, cells were fixed for 15 min in 4% formaldehyde solution and stored in PBS overnight. A 1 mL of 5% silver nitrate solution (5 g silver nitrate, 100 mL deionized H_2_O, kept in dark) was added to each sample well and exposed to ultraviolet (UV) light for 20 min. Samples were washed thrice in deionized water. A 1-mL 5% sodium thiosulphate solution (5 g sodium thiosulphate, 100 mL deionized H_2_O, stored in dark) was added for 10 min, and samples were washed as described above. A 1-mL counterstain solution (0.1 g nuclear fast red, 5 g aluminium sulphate in 100 mL deionized H_2_O, boiled for 5 min and filtered) was added for 3 min. Samples were washed as described above and finally rinsed in 70% ethanol. Digital images of PCL discs were captured and saved for further analysis. The whole procedure was carried out at 37°C to maintain gel integrity. The hydrogel was washed away during the staining protocol and therefore is not imaged.

### Quantitative polymerase chain reaction

Samples were harvested after 28 days of culture in triplicate by transferring hydrogels to a 2 mL Eppendorf tube and adding 1 mL of TRIZOL reagent and incubating at RT for 10 min. Cells on PCL substrates were removed by trypsinization and cell pellet was added to corresponding hydrogel TRIZOL solution. Samples were stored at −80 °C until RNA isolation. RNA was isolated using RNAeasy micro kit according to the manufacturer’s protocol (Qiagen, UK). RNA pellets were solubilized in RNase-free water and assessed for concentration and purity with measured absorbance at 230 nm, 260 nm (nucleic acids) using a NanoDrop ND 100 spectrometer (Thermo Scientific, USA). Reverse transcription was carried out on extracted RNA using an Omniscript Reverse Transcription kit (Qiagen) according to the manufacturer’s instructions, with Random Primers (Invitrogen) and RNAsin (Promega, USA). Quantitative polymerase chain reaction (qPCR) was carried out using a qPCR detection system (model 7500; Applied Biosciences, UK) by the SYBR green method. Expression of SOX9 (Table 2) was tested, and glyceraldehyde 3-phosphate dehydrogenase (GAPDH) expression was used as a reference gene to normalize all data. Relative gene (RQ) expression values were automatically calculated by the delta delta CT method. Statistical analysis first determined that GAPDH did not vary under test conditions (one-way analysis of variance (ANOVA)). Cycle threshold values were then converted from logarithmic to linear scale (2^ΔΔ-CT^) for further analysis.

**Table 2.** Primers used for qPCR.

Primer	Forward	Reverse
GAPDH	TCAAGGCTGAGAACGGGAA	TGGGTGGCAGTGATGGCA
SOX9	AGACAGCCCCCTATCGACTT	CGGCAGGTACTGGTCAAACT

GAPDH: glyceraldehyde 3-phosphate dehydrogenase; qPCR: quantitative polymerase chain reaction.

### Statistical analysis

For the analysis of gene expression, the 2^ΔΔ-CT^ method was used.^28^ Statistical analysis was carried out using the Tukey Kramer multiple-comparisons post-test ANOVA. Relative transcript levels expressed as the mean ± standard deviation for plotting on graphs (n = 3 for each condition). doi: http://dx.doi.org/10.5525/gla.researchdata.399

## Results

**Figure 1.**
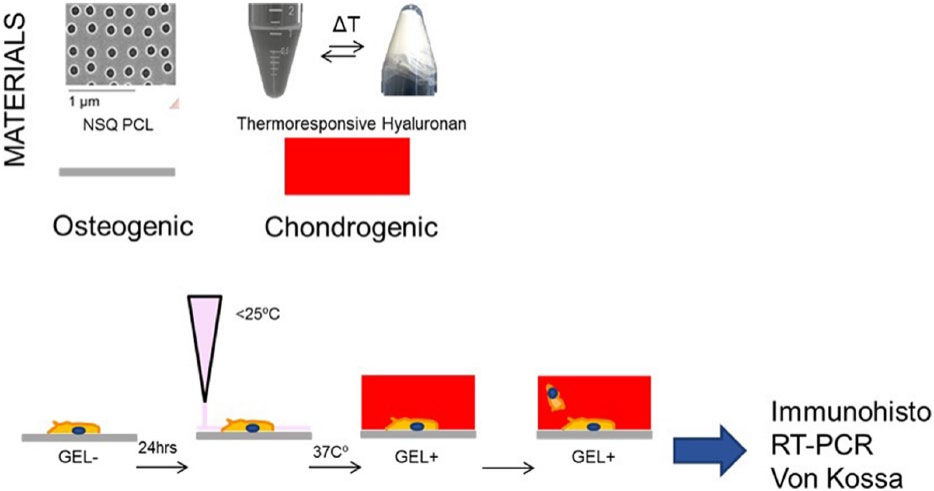
Schematic of basic in vitro experimental set up.

Unless stated, experiments were set up as depicted in the scheme in Figure 1, with MSCs seeded onto the flat control or NSQ nanotopographical for 24 h before setting the gel on top of the cells.

### Materials

**Figure 2.**
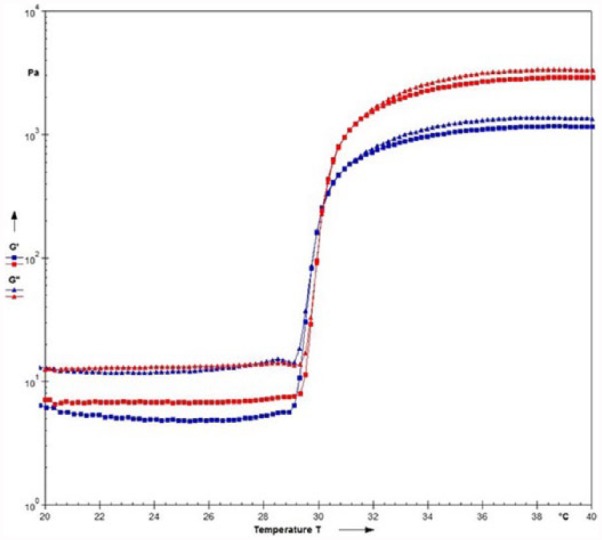
Rheological features of the gel with (blue symbols) and without (red symbols) hMSCs. Cells were seeded at 20 × 10^6^ cell/mL. Squares represent storage moduli, and triangles represent loss moduli. Cell presence has a minor impact on the rheological properties.

Thermoresponsive hyaluronan derivatives formulation used for this study had an average degree of substitution value of 6.42% ± 0.40% as measured by ^1^H NMR, an average storage modulus (G′) value of 0.46 ± 0.22 Pa at 25 °C and 1090 ± 660 Pa at 35 °C. To assess the influence of hMSCs on the stability and rheological properties of the hydrogel formulation, 20 million hMSCs were seeded encapsulated into 1 mL of hyaluronan derivative reconstituted at 15% w:v in PBS and the rheological profile measured (Figure 2). The gelation temperature was not influenced by the presence of cells, while the final storage modulus value decreased from 6.7 to 4.8 Pa at 25°C and from 1100 Pa down to 1000 Pa at 35°C.

### Morphological analysis

**Figure 3.**
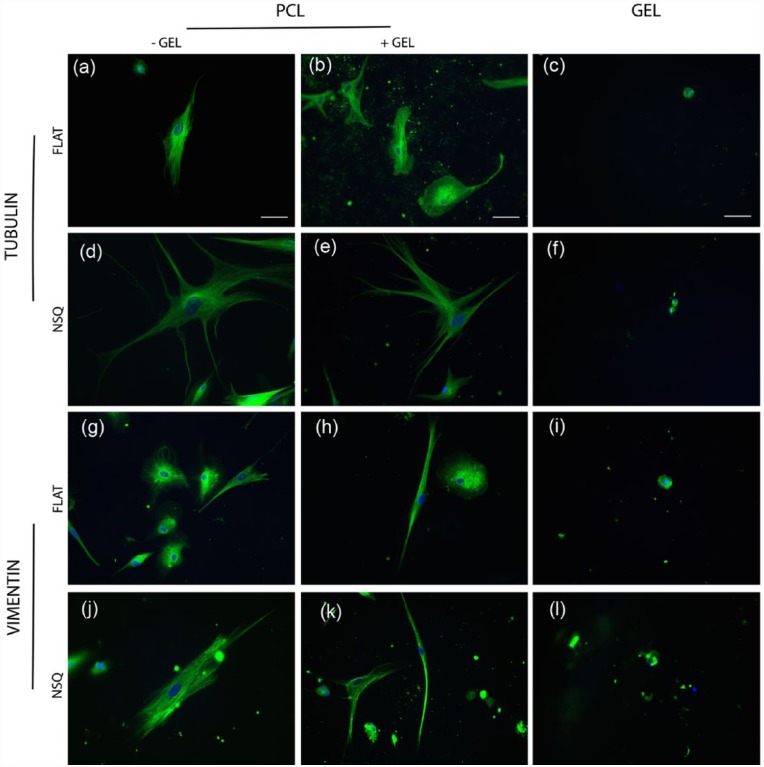
Cytoskeletal morphology analysis of MSCs cultured on FLAT and NSQ topography with and without addition of Hyal hydrogel. Images of immunostained MSCs cultured for 5 days on PCL surfaces of (a–c, g–i) FLAT or NSQ (d–f, j–l) topography, with and without addition of hydrogel. Cells exhibit varied morphology typical of several mesenchymal lineages on FLAT surfaces +/− gel, where NSQ leads to well-spread polygonal cells typical of osteoblast morphology. Note rounded morphology, typical of chondrogenesis, in hydrogels cultured with either FLAT or NSQ surfaces. Green is (a–f) tubulin, (g–l) vimentin, and blue is DAPI nuclear stain. Scale bar is 50 µm.

**Figure 4.**
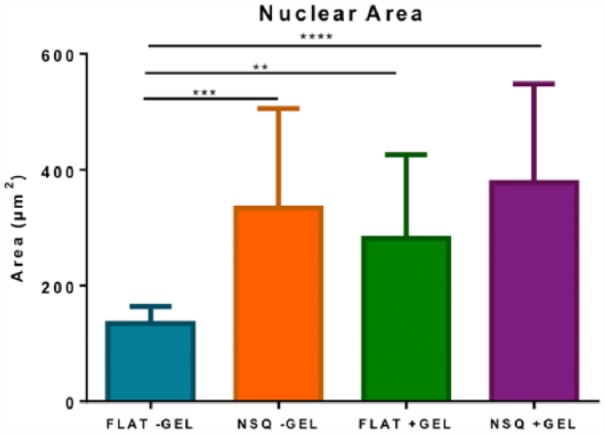
Nuclear area of hMSCs cultured on FLAT +/−GEL and NSQ +/−GEL for 5 days. NSQ topographies consistently lead to a larger nuclear area, indicative of increased cellular spreading associated with osteoblastic morphologies. The change in matrix elasticity upon addition of the hydrogel also led to a lesser yet significant increase in nuclear area on FLAT surfaces. Data presented is mean nuclear area ±SD. Comparison was done by ANOVA **p < 0.01, ***p < 0.001, ****p < 0.0001, n = 20.

MSCs cultured for 5 days on FLAT or NSQ patterned PCL with and without the addition of the hydrogel were first subject to immunofluorescence microscopy in order to analyse differences in cellular morphology between the different materials. Samples were fluorescently stained for either β3-tubulin or vimentin alongside a nuclear stain. The FLAT control surfaces exhibited great variation in cellular morphology within samples, with most cells spindle-shaped typical of fibroblast morphologies, alongside more stellate or rounded morphologies more typical of osteogenic or chondrogenic/adipogenic lineages (Figure 3(a) and (b) (tubulin) and Figure 3(g) and (h) (vimentin)), whereas NSQ surfaces consistently led to well spread, polygonal cells with large nuclei (Figure 4), typical of osteoblastic morphologies (Figure 3(d) and (e) (tubulin) and Figure 3(j) and (k) (vimentin)). More organized tubulin microtubule and vimentin intermediate filament networks were observed in larger, polygonal cells on the NSQ surfaces (Figure 3(d) and (e) (tubulin) and Figure 3(j) and (k) (vimentin)). Within the hyaluronan hydrogel, a small number of cells were observed. Cells in the hydrogel exhibited rounded morphologies of ~10–25 µm in diameter typical of chondrogenic morphology, and this was maintained throughout samples regardless of underlying surface topography.

### Transcription factor expression analysis

Following the analysis of MSC morphology, expression of phosphorylated runt-related transcription factor type 2 (pRUNX2), a marker for osteogenic differentiation, and sex-determining region Y-box 9 (SOX9), a transcription factor involved in chondrogenic differentiation, were observed by immunofluorescence microscopy in MSCs cultured for 5 days on FLAT or NSQ patterned PCL with and without the addition of the hydrogel.

**Figure 5.**
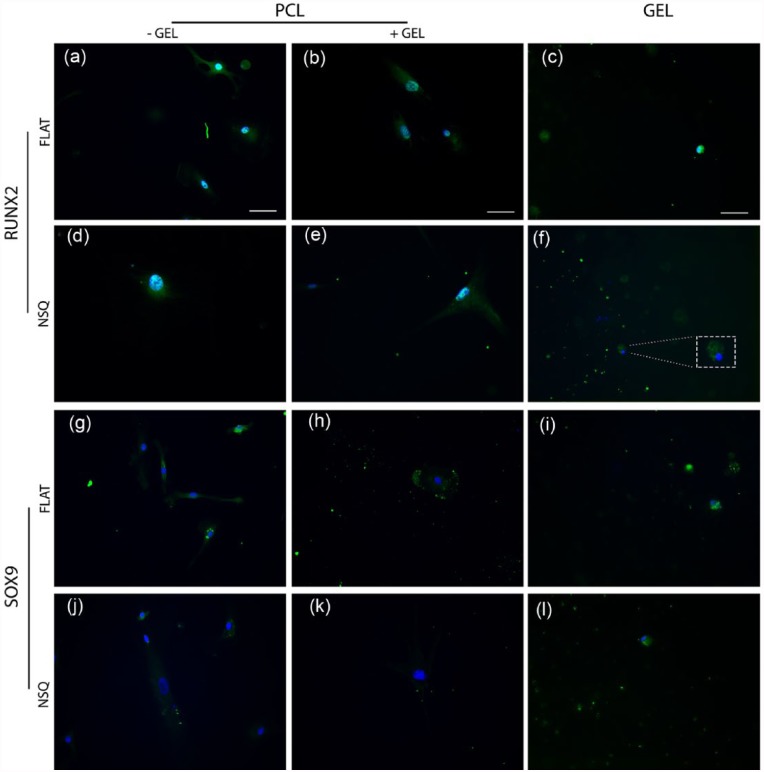
Osteogenic and chondrogenic transcription factor expression analysis of MSCs cultured on FLAT and NSQ topography with and without the addition of Hyal hydrogel. Images of immunostained MSCs cultured for 5 days on PCL of (a–c, g–i) FLAT or (d–f, j–l) NSQ topography, with and without addition of hydrogel. (a–f) Osteogenic marker phosphorylated RUNX2 expression, note large nuclear area on NSQ surfaces with high nuclear expression and localization. Note expression in rounded cells in hydrogel. (g–l) Chondrogenic marker SOX9 expression, note cytoplasmic expression in FLAT samples compared to very low levels detected on NSQ, with cells in hydrogels showing clear positive expression. Green is (a–f) phosphoRUNX2, (g–l) SOX9, and blue is DAPI nuclear stain. Scale bar is 50 µm. Turquoise colour (blue/green overlay) = co-localization.

RUNX2 expression on FLAT surfaces was abundant in both the nucleus and in the perinuclear region of the cytoplasm (Figure 5(a) and (b)), whereas on NSQ surfaces RUNX2 appears highly abundant in the nucleus with only negligible detection in the perinuclear region (Figure 5(d) and (e), co-localization shown as turquoise colour (blue/green overlay)). Also, there were notably larger nuclei observed in the NSQ populations compared with control FLAT populations (more examples of MSC nuclei on NSQ in Supplementary Figure 2). It should also be noted that RUNX2 is also an indicator of late chondrogenesis;^29,30^ and positive RUNX2 expression was detectable in cytoplasm (not in the nuclei) of MSCs that had migrated into the hydrogel regardless of underlying PCL surface topography (Figure 5(c) and (f)).

SOX9 expression was detected on FLAT surfaces at low levels in some cells throughout the sample but was located in the cytoplasm only (Figure 5(g) and (h)). In MSCs on the NSQ surfaces, SOX9 expression was mainly negligible (Figure 5(j) and (k)). Consistent with RUNX2 expression, SOX9 was abundantly present in cells that had migrated into the hydrogels regardless of the underlying PCL surface topography (Figure 5(i) and (l)).

### Matrix maturation and mineralization

**Figure 6.**
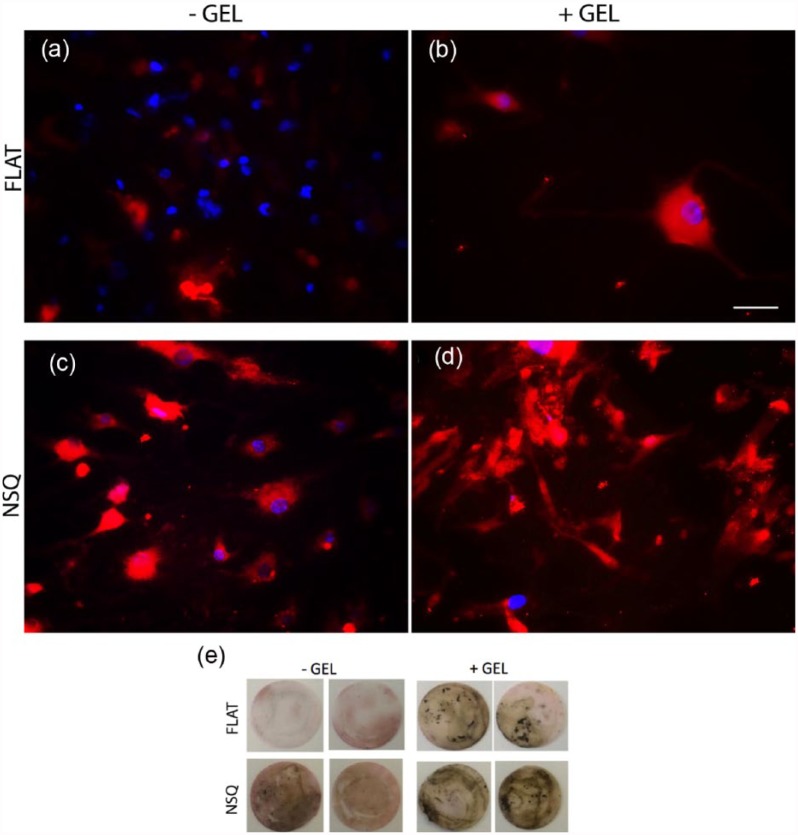
MSC mineralization analysis following long-term culture. (a–d) RGB images of immunostained MSCs cultured for 28 days on PCL surfaces with (a, b) FLAT or (c, d) NSQ topography, (b, d) with and (a, c) without the addition of hydrogel. Samples were stained for osteocalcin secretion. (e) MSCs cultured on PCL for 28 days and von Kossa stained. Note increase in positive staining in all NSQ compared to FLAT, with slight increase also observed on FLAT +GEL. Green is osteocalcin, and blue is DAPI nuclear stain. Scale bar is 100 µm.

MSCs were cultured on FLAT and NSQ surfaces with and without hydrogel for 28 days and immunofluorescence was used to detect expression of the bone-specific ECM protein osteocalcin (OCN), an osteoblast differentiation marker. On the FLAT surfaces without gel, only very low levels of OCN were detected (Figure 6(a)). With the addition of gels, OCN expression increased notably (Figure 6(b)). OCN levels were, however, high on the NSQ samples without the hydrogel (Figure 6(c)) and this expression of OCN increased further with hydrogel addition (Figure 6(d)).

Further to this, MSCs that had been cultured for 28 days were assessed for mineralization via von Kossa staining (stain for calcium deposits present in bone mineral, therefore suggestive of osteogenic differentiation), in which the trend observed was consistent with the immunofluorescence analysis with maximal expression seen in cells cultured on NSQ with gels on top (note that the gel was removed during staining) (Figure 6(e)).

## Discussion

This study investigated the use of a chondropermisive hyaluronan hydrogel in combination with a well-defined topography presented on a biodegradable, biocompatible polymer as a method to produce a tissue engineered cartilage-bone interface from a single multipotent cell source.

The NSQ topography provides targeted osteogenesis from MSC populations, while the FLAT control surface results in uncontrolled heterogeneous differentiation. This is in agreement with published data.^11^ Cells cultured on NSQ consistently had a well-spread, polygonal morphology with notably increased nuclear size in comparison to those cultured on control surfaces. It is now understood that the NSQ arrangement is able to promote osteogenesis by encouraging this well-spread morphology. Through integrin receptor–related signalling^31^ and bone morphogenetic protein 2 (BMP2) signalling,^32^ RUNX2 is phosphorylated (activated) and thus the transcription of osteoblast-specific genes essential for bone homeostasis occurs.^33^ Furthermore, changes in nucleus size in cells on NSQ has been implicated in changing chromosomal positioning and hence direct cellular mechanotransduction.^25,34^ When cultured with the hydrogel in place, NSQ surfaces continued to direct osteogenesis of MSCs on the surface, whereas FLAT surfaces displayed evidence of an increase in a rounded cell population on the surface. This can be speculated to suggest that cues presented by the hydrogel in three-dimensional (3D) culture predominate other mechanical or chemical cues presented by the FLAT surfaces but not the topographical cues presented by the NSQ surfaces. This is perhaps logical as the cell–cell mimicking effects of hyluronan have been previously implicated in chondrogenesis,^35^ through interaction with the CD44 antigen.

Previous studies have confirmed the viability of culturing MSCs in the hydrogel and also presented strong evidence of chondropromotive environment provided by the hyaluronan-based hydrogel,^15,36^ with a further study investigating the biocompatibility in vivo.^37^ The addition of cells to the hydrogel led to a decrease of storage modulus after the transition. This is to be expected because the gelation mechanism is based on non-covalent interactions prone to disruption by the presence of MSC, especially at such a high concentration. However, the gelation temperature remained unvaried and the cell-containing hydrogel underwent a >200-fold increase of storage modulus in a very narrow temperature window. In agreement with the previous findings, we found cells cultured in the hydrogel were consistently rounded, typical of chondrocyte morphology with clear expression of chondrogenic marker SOX9. Although not statistically significant, due to a large volume of hydrogel and small volume of cells introducing variability, gene expression analysis shows SOX9 detection in the gel (Supplementary Figure 3). It is suggested that the lack of degradation sensitive sites presented in the hydrogel network restricts cellular spread, promoting this rounded morphology and thus directing chondrogenic differentiation.^3,15^ Furthermore, there is a lack of integrin-specific ligands in the gel. In the same study, hMSCs cultured in the hydrogel underwent chondrogenic differentiation even when cultured in osteogenic media.^15^ It is noteworthy that HA is a native component of cartilage and as indicated in this, and previous studies, it may be responsible for maintaining the chondrocyte phenotype.

When considering orthopaedic applications of the construct, injection of the hydrogel into subchondral defects in rabbit highlighted clinical transferability of the gel itself. In the study by D’Este et al.,^37^ biocompatibility and ease of use were confirmed, as the gel was injected into the site of the defect. Gel shearing from the nanotopographical surface could present a potential limitation of this construct upon scaling-up. However, the 2016 study validated retention of the gel in a partially weight-bearing osteochondral defect within a moving synovial joint for up to 12 weeks; it was also noted here that the implanted gels had lost their reversibility upon long-term harvesting.^37^

This study highlights that differential tuning of chemical, physical and mechanical properties of the extracellular environment can lead to the targeted differentiation of cells down different tissue lineages within the same culture. Two materials were used for in vitro culture of MSCs, both of which have previously been fully characterized.^5,15^ Here, we introduce the possibility to create interfaces capable of directing anisotropic cell and potentially tissue growth, which can have important implications in complex tissue engineering. Analysis confirmed that when put together, the materials retained their abilities to direct differentiation down two distinct lineages, as expected, NSQ topography consistently led to osteogenesis and we were able to confirm targeted chondrogenesis of cells that migrated from the surface into the hydrogel. This novel system combines two simple materials with different differentiation capacities and cells from a single source to create a platform with the capability to sustain the growth of two tissue types in culture. This proof-of-concept system highlights that multi-compartmental material systems that control spatial differentiation of MSCs can be made, but the current system is perhaps more suited to drug testing than orthopaedic use due to its mechanical characteristics that need to be developed. It is envisioned that creating more biomimetic grafts through complex tissue engineering techniques as such could be exploited to improve success of current approaches.

## Supplementary Material

Supplementary material
