# Supplementary material for: Bone and cartilage differentiation of a single stem cell population driven by material interface
Source: J Tissue Eng. 2017 May 15;8:2041731417705615. doi: 10.1177/2041731417705615 (PMC5438107; doi:10.1177/2041731417705615)
Supplement: Supplementary material [file Supplementary_information.docx]

Supplementary information for:

# **Bone and cartilage differentiation of a single stem cell population driven by material interface.**

Author names

Hannah Donnelly^1^, Carol-Anne Smith^1^, Paula E Sweeten^1^, Nikolaj Gadegaard^2^, R.M. Dominic Meek^3^, Matteo D'Este^4^, Alvaro Mata^5,6^, David Eglin^4^, Matthew J Dalby^1^

Affiliations:

^1^Centre for Cell Engineering, University of Glasgow, Glasgow, U.K.

^2^Division of Biomedical Engineering, University of Glasgow, Glasgow, U.K.,

^3^Department of Orthopaedics, Southern General Hospital, Glasgow, U.K.

^4^AO Research Institute Davos, Davos, Switzerland

^5^Institute of Bioengineering, Queen Mary University of London, London, U.K.

^6^School of Engineering and Materials Science, Queen Mary University of London, London, U.K.

Corresponding author:

Hannah Donnelly, Centre for Cell Engineering, University of Glasgow, Joseph Black Building, University of Glasgow, Glasgow, G12 8QQ, U.K.

Email: H.donnelly.1@research.gla.ac.uk

**
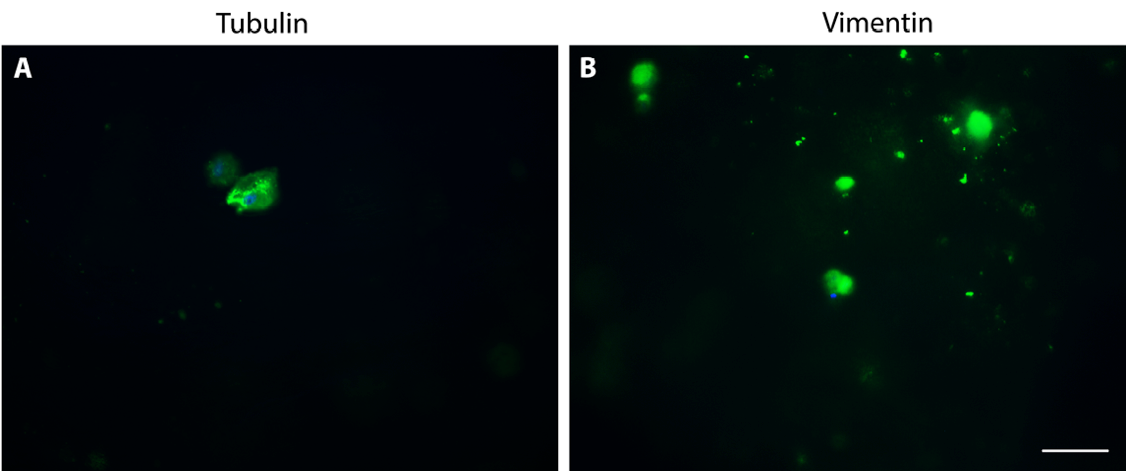
**

Supplementary figure 1. Morphology of mesenchymal stem cells (MSC) seeded directly into the hydrogel (1 x 10^4^ cells/mL). Images of cytoskeletal immunostained MSC populations cultured for 5 days in thermoresponsive hyaluronan hydrogels. Note rounded morphology of cells. Green is (a) tubulin, (b) vimentin, blue is DAPI nuclear stain. Scale bar is 50 µm.


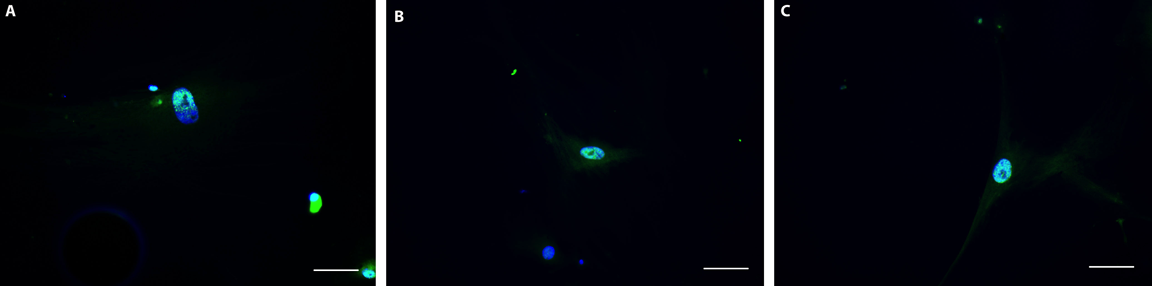


Supplementary figure 2. Nuclear morphology of RUNX2 positive nuclei on NSQ (-gel). Images of immunostained mesenchymal stem cells (MSC) cultured for 5 days on 5 days on NSQ showed consistently large nuclear morphology with highly nuclear localized phosphorylated RUNX2 expression. Cells with smaller nuclear area tended to show less expression (see (b)). Green is phosphorylated RUNX2, blue is DAPI nuclear stain. Scale bar is 50 µm.

Supplementary figure 3.qPCR for SOX9 in mesenchymal stem cells (MSC) cultured on flat control and on flat control overlaid with hydrogel. SOX9 expression increases with cell migration into the gel. 28 days culture, n = 3.
